# Review of the neglected tropical diseases programme implementation during 2012–2019 in the WHO-Eastern Mediterranean Region

**DOI:** 10.1371/journal.pntd.0010665

**Published:** 2022-09-29

**Authors:** Supriya Warusavithana, Hoda Atta, Mona Osman, Yvan Hutin

**Affiliations:** Department of Universal Health Coverage/Communicable Diseases Prevention and Control, WHO EMRO Eastern Mediterranean Regional Office, World Health Organization, Cairo, Egypt; Faculty of Science, Ain Shams University (ASU), EGYPT

## Abstract

**Introduction:**

The 2012–2020 WHO NTD roadmap set targets for control, elimination, and eradication of neglected tropical diseases (NTDs). It recommends 5 strategies, out of which preventive chemotherapy (PC) and intensified disease management were key to achieve targets. WHO estimated that globally, between 2012 and 2019, the number of persons affected by NTDs decreased from nearly 2.1 to 1.7 billion people. We analysed the situation of NTDs in the WHO Eastern Mediterranean Region (EMR) in 2020 to assess the progress with the 2012–2020 roadmap and to identify gaps.

**Methods:**

We reviewed data repositories of national data sources for 2012 to 2019 including the Global Indicator Data Platform for Sustainable Development Goals, the Global Health Observatory data repository, the WHO PC databank, and the EMR data repository. We allocated countries a Red-Amber-Green (RAG) rating based on standardized criteria, on progress and current situation of each of 11 priority NTDs.

**Results:**

All 22 countries in EMR were affected by 1 or more autochthonous or imported NTDs. In 2019, WHO estimated that in EMR, 78 million people required interventions for NTDs, a 38% decline compared with 2012. Twelve of 22 countries needed priority public health action (i.e., red) for 1 or more NTD. Of these, Sudan needed priority public health action for 6 NTDs and Yemen for 5. Eleven countries also needed priority public health action for cutaneous leishmaniasis, and 5 countries for rabies and trachoma. Visceral leishmaniasis is on the increase in Afghanistan, Libya, Syria, and Yemen.

**Conclusion:**

Since the first roadmap of NTDs in 2012, the EMR has made a substantial progress. Nevertheless, many challenges remain in the prevention and control of NTDs. EMR needs a regional approach to control NTDs in countries most affected and a coordinated strategy to stop the continuing increase of cutaneous leishmaniasis and a possible resurgence of visceral leishmaniasis.

## Introduction

The World Health Organization (WHO) estimated that in 2000, neglected tropical diseases (NTDs) affected nearly 2 billion people worldwide [1]. In 2013, a study suggested that they caused approximately 152,000 deaths and nearly 48 million disability adjusted life years (DALYs) globally [2]. NTDs are diseases of poverty that impose a devastating human, social, and economic burden on people living in predominantly tropical and subtropical areas, among the most vulnerable, marginalized populations [3].

In 2012, WHO published a roadmap to overcome the impact of NTDs through the identification of global and regional targets for the eradication, elimination, and control of NTDs by 2020. The roadmap listed 2 major strategic interventions to achieve these targets: preventative chemotherapy and intensified disease management. Other interventions including vector and intermediate host control, veterinary public health, and provision of safe water and sanitation were also referred to as key for the effective prevention and control of NTD [4]. In 2021, WHO proposed a new NTD road map for 2021–2030, with a new model of operation to achieve Sustainable Development Goal (SDG) targets on NTD [3]. The new road map encourages 3 fundamental shifts in the approach to tackling NTDs. First, we should increase accountability for impact by using impact indicators instead of process indicators and accelerate programmatic action. Second, we should move away from siloed, disease-specific programmes by mainstreaming programmes into national health systems and intensifying cross-cutting approaches with adjacent sectors, for example, education, WASH, agriculture, environment,

livestock, wildlife (One Health approach) centred on the needs of people and communities, thereby improving cost–effectiveness, coverage and geographical reach of programmes. Third, we should change operating models and culture to facilitate greater ownership of programmes by countries [3].

In the WHO’s Eastern Mediterranean Region (EMR), some NTDs, such as leishmaniasis, are endemic in most countries in the region. Other diseases, such as onchocerciasis and schistosomiasis, affect only some countries or are of low prevalence [5]. As such, NTD programmes vary greatly in their organization in different countries of the region, depending on disease’s geographical distribution, available capacity, and allocated resources. This can range from standalone vertical programmes like National Leprosy Control Programme in Morocco [6], integrated NTD programmes like in Yemen [7], or to integration with other diseases like National Malaria and Leishmaniasis Control Programme in Afghanistan [8]. To ensure its successful implementation in the region, the essence of the new global roadmap [3] will need to be adapted to the context of Member States in EMR and to be guided by the current epidemiology and gains achieved through implementation of the 2012 Roadmap. Thus, we analysed the situation of the NTDs in the Member States in EMR to assess progress and identify gaps in the prevention and control of NTDs to inform the development of the 5-year regional implementation plan to accelerate the global NTD Roadmap 2021–2030 [3].

## Methods

### Data sources

We used data from 4 data repositories to inform the review of NTDs in EMR countries for all available years in 2012 to 2019 (Table 1).

**Table 1:** List of NTDs, their indicators, and data repositories used.

Disease/Goal	Indicator(s)	Repository
SDG 3	Number of people requiring interventions against NTDs	Sustainable Development Goals Indicators Data Platform https://unstats.un.org/sdgs/dataportal/
SDG 6	Proportion of population using safely managed drinking water services	
	Proportion of population using (a) safely managed sanitation services and (b) a handwashing facility with soap and water	
Leishmaniasis (cutaneous)	Status of endemicity of cutaneous leishmaniasis	Global Health Observatory https://www.who.int/data/gho/data/themes/neglected-tropical-diseases
	Number of cases of cutaneous leishmaniasis reported	
Leishmaniasis (visceral)	Status of endemicity of visceral leishmaniasis	
	Number of cases of visceral leishmaniasis reported	
Leprosy	Number of new leprosy cases	
	Number of leprosy cases registered for treatment (prevalence)	
Rabies	Reported number of human rabies deaths	
Yaws (endemic treponematodes)	Status of yaws endemicity	
Lymphatic filariasis	Status of MDA	Preventive Chemotherapy Data Portal https://www.who.int/data/preventive-chemotherapy/
	National coverage (%)	
	Number of intervention units requiring PC	
	Number of IUs covered	
	Geographical coverage (%)	
	Total population of IUs	
	Reported number of people treated	
	Programme coverage (%)	
	National coverage (%)	
Onchocerciasis	Status of endemicity	
	National coverage (%)	
STHs	National coverage (%) of SAC	
Schistosomiasis	Status of schistosomiasis endemic countries	
	Number of SAC to be treated	
	National coverage (%) of SAC	
Trachoma	Status of elimination of trachoma as a public health problem	
Mycetoma	Mycetoma	EMR Regional Health Observatory https://rho.emro.who.int/Indicator/TermID/64

EMR, Eastern Mediterranean Region; IU, Implementation Unit; MDA, mass drug administration; NTD, neglected tropical disease; PC, preventive chemotherapy; SAC, school-aged children; SDG, Sustainable Development Goal; STH, soil-transmitted helminthiases.

#### Global indicator data platform for Sustainable Development Goals (SDGs)

The Inter-Agency and Expert Group on SDG Indicators developed this framework that includes 231 unique indicators. We downloaded and analysed data for 3 relevant indicators: 1 for Goal 3 (healthy lives and promote well-being for all at all ages) and 2 for Goal 6 (availability and sustainable management of water and sanitation for all). First, the number of people requiring interventions for NTDs, defined as the average number of people requiring preventive chemotherapy (PC) and individual treatment and care, for at least 1 NTD (3.3.5). Second, (6.1) the proportion of population using safely managed drinking water services, by urban/rural status. Third, (6.2), the proportion of population using safely managed sanitation services, using handwashing facility with soap and water and practicing open defecation.

#### Global Health Observatory data repository

The WHO Global Health Observatory is a data repository of indicators and health-related statistics including NTDs collected from Member States. We selected NTDs based on availability of data and downloaded data for leishmaniasis (cutaneous and visceral), leprosy, rabies, and endemic treponematoses (Table 1).

#### Preventive chemotherapy (PC) data portal

The WHO data repository provides data for NTDs for which the main public health strategy is PC and includes lymphatic filariasis, onchocerciasis, soil-transmitted helminthiases (STH), schistosomiasis, and trachoma. Indicators include the population requiring treatment, population receiving treatment, and coverage figures. National NTD programmes submit epidemiological data from surveys and routine surveillance, request medicines, and report on PC implementation through a standardized set of forms called the Joint Application Package. WHO then reviews these forms, facilitates medicine supply, reports on progress, and publishes PC indicators in the portal. We extracted indicators to analyze coverage figures (Table 1).

#### Eastern Mediterranean Regional Health Observatory (http://rho.emro.who.int/)

The repository provides the number of reported cases of mycetoma.

### Red-Amber-Green (RAG) status

We used the RAG ratings, also known as “traffic lighting,” which have been used to summarize performance indicators. We constructed criteria for RAG status using targets outlined in the 2012–2020 road map for NTD [WHO 2012] (Table 2). We then assigned a RAG status to each country for each relevant priority NTD (Table 3). Red meant that priority action was needed, amber that continued action was needed, green meant that targets have been achieved, and white meant that NTD was either nonendemic or did not require PC. We limited our review to NTDs for which data are available for EMR countries.

**Table 2:** List of NTDs with targets extracted from WHO Roadmap for 2012–2020 with priority public health approaches (intensified disease management or PC) and criteria for RAG status.

Priority public health strategy		Targets outlined in WHO Roadmaps for NTD 2012–2020	Criteria for RAG rating for each disease		
	Disease		Red	Amber	Green
**Intensified disease management**	Dracunculiasis (Guinea worm)	Eradication by 2015	Continuing need for interventions including strong surveillance	Awaiting certification of eradication	Certified eradication
	Endemic treponematoses	Eradication by 2020	Ongoing endemicity	Previously endemic	Certified eradication
	Leprosy	Global elimination by 2020	Prevalence ≥1 registered case/10,000 population	Prevalence <1 case registered case/10,000 population	0 registered cases
	Leishmaniasis (visceral)	Regional elimination in SEAR	≥10 autochthonous cases reported last available year and increasing when compared with 2012 with interventions	≥10 autochthonous cases reported last available year and declining when compared to 2012	<10 autochthonous cases reported at last available year
	Leishmaniasis (cutaneous)	70% of all cases detected and at least 90% of all detected cases treated in the EMR by 2015	≥1,000 autochthonous cases reported last available year and increasing when compared with 2012	<1,000 and ≥100 autochthonous cases reported last available year and declining when compared to 2012	<100 autochthonous cases reported at last available year
	Rabies	Regional elimination in 3 other regions by 2020	Average number of deaths per year reported (2012–2018) ≥5.0	Average number of deaths per year reported (2012–2018) <5.0	No deaths reported 2012–2018
	Mycetoma	Not applicable	≥100 cases reported in any 1 year since 2016	<100 cases reported in any 1 year since 2016	0 cases reported in all years since 2016
**PC**	Lymphatic filariasis	Global elimination by 2020	Preventative chemotherapy required	MDA stopped	Validated elimination as a public health problem
	Onchocerciasis	Country elimination in Yemen by 2015	Intervention required to be implemented	Intervention scaled up (last year)	Verified elimination of transmission
	Schistosomiasis (bilharzia)	Regional elimination in EMR by 2015	National treatment coverage <75% (last available year)	National treatment coverage >75% (last available year)	No PC required
	STH	75% of preschool and school-aged children in need of treatment regularly treated and in all countries by 2020	National treatment coverage <75% (last available year)	National treatment coverage >75% (last available year)	No PC required
	Trachoma	Global elimination by 2020	Continued intervention needed	May need intervention or awaiting validation of elimination as a public health problem	Validated elimination as a public health problem

EMR, Eastern Mediterranean Region; MDA, mass drug administration; NTD, neglected tropical disease; PC, preventive chemotherapy; RAG, Red-Amber-Green; SEAR, South-East Asia Region; STH, soil-transmitted helminthiases; WHO, World Health Organization.

**Table 3:** RAG status of priority NTD by country, EMR, 2012–2020.

Country	Leishmaniasis (cutaneous)	Leishmaniasis (visceral)	Leprosy	Rabies	Trachoma	Helminthiases	Mycetoma	Schistosomiasis	Onchocerciasis	Lymphatic filariasis	Guinee Worm	Endemic treponematoses
**Sudan**												
**Yemen**												
**Somalia**												
**Egypt**												
**Iraq**												
**Pakistan**												
**Afghanistan**												
**Syria**												
**Morocco**												
**Iran**												
**Libya**												
**Tunisia**												
**Saudi Arabia**												
**Djibouti**												
**Palestine**												
**Jordan**												
**Lebanon**												
**Qatar**												
**Oman**												
**Kuwait**												
**UAE**												
**Bahrain**												
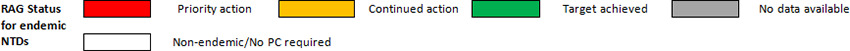												

EMR, Eastern Mediterranean Region; NTD, neglected tropical disease; PC, preventive chemotherapy; RAG, Red-Amber-Green.

## Results

### Overview of NTDs in EMR

The WHO Roadmap (2012–2020) identified public health targets for 17 different NTDs. Our review covered NTDs endemic to the EMR and their specific targets (Table 2). All 22 countries in EMR were affected by 1 or more NTD (autochthonous or imported). Three countries (Somalia, Sudan, and Yemen) were affected by 7 or more NTDs. Most countries (21) were affected by leprosy followed by leishmaniasis (18 countries for the cutaneous and visceral forms) and rabies (12 countries). Twelve countries required priority public health action (i.e., a red RAG rating) for 1 or more NTD. Of these, 2 countries required priority public health action for 5 or more NTDs (6 in Sudan and 5 in Yemen). Cutaneous leishmaniasis required priority health action (i.e., red RAG rating) in 11 countries followed by trachoma and rabies (5 each; Table 3). An estimated 78 million people in EMR (12% of the population) required interventions for NTDs in 2019. Most of these (82%) resided in 4 countries (Pakistan, Afghanistan, Yemen, and Sudan). Between 2012 and 2019, the estimated number of people requiring interventions against NTDs in EMR declined from 124.9 to 77.8 million (38%). Of the 22 countries in the region, 14 reported a decline, largest in Sudan (from 30.8 to 12.0 million) and Egypt (from 24.0 to 6.9 million). Afghanistan (from 14.0 to 16.2 million), Pakistan (from 31.5 to 31.7 million), and Syria (from an average of 53,000 per year until 2016 to 2.4 million in 2019) reported increases (S1 and S2 Tables).

### Review of NTDs controlled by intensified disease management

#### Diseases targeted for eradication

Two NTDs were targeted for eradication: dracunculiasis and endemic treponematoses. Sudan was the only country in EMR affected by dracunculiasis. The last reported case was in 2013 and by 2020, the country was at the precertification stage of being declared dracunculiasis-free pending review by the International Commission for the Certification of Dracunculiasis Eradication (ICCDE). Endemic treponematoses were reported in Somalia in the past, but the 2020 status of the country has been reported as previously endemic.

#### Diseases targeted for elimination

**Fig 1:**
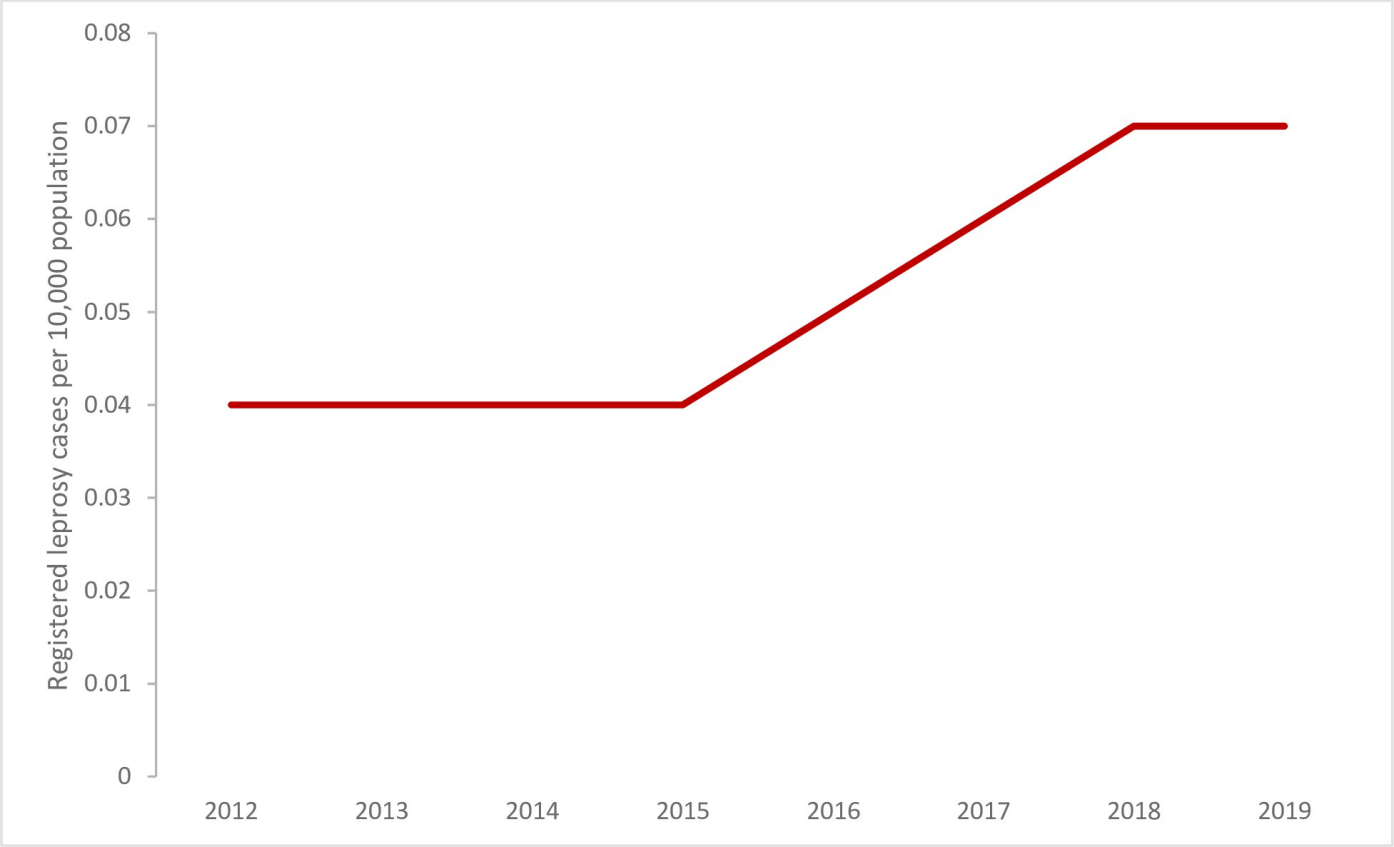
The number of leprosy cases registered for treatment per 10,000 population (prevalence) in the Eastern Mediterranean Region, 2012–2019, Global Health Observatory data repository (https://apps.who.int/gho/data/node.main.A1638?lang=en).

Leprosy has been targeted for elimination as a public health problem (defined as less than 1 case registered for treatment per 10,000 population [4]). In 2019, the number of registered cases for treatment was below the target for elimination in EMR (0.07/10,000) (Fig 1) in all countries except Somalia (1.54/10,000) (S3 Table). Five countries (Egypt, Pakistan, Somalia, Sudan, and Yemen) reported 98% of the 4,944 leprosy cases registered for leprosy treatment in EMR in 2019. Of the remaining 17 countries, 7 reported between 1 to 25 cases, 4 reported no cases, and 6 did not report leprosy data in 2019. The total cases registered for treatment in 2019 represented a 69% increase comparing with the leprosy cases reported in 2012 (2,928 reported by 19 countries; Fig 1), mainly because of increased case detection due to intensified efforts from Somalia (from 139 cases in 2012 to 2,455 in 2019; S3 Table).

#### Cutaneous leishmaniasis

In 2020, leishmaniasis was endemic in all except 3 countries in EMR (Bahrain, Qatar, and United Arab Emirates). In 2019, the EMR accounts for 80% of cases of cutaneous leishmaniasis reported globally to WHO. In 2019, the region also reported 24.9% of the global burden of visceral leishmaniasis (all endemic WHO regions accounting for the same share).

**Fig 2:**
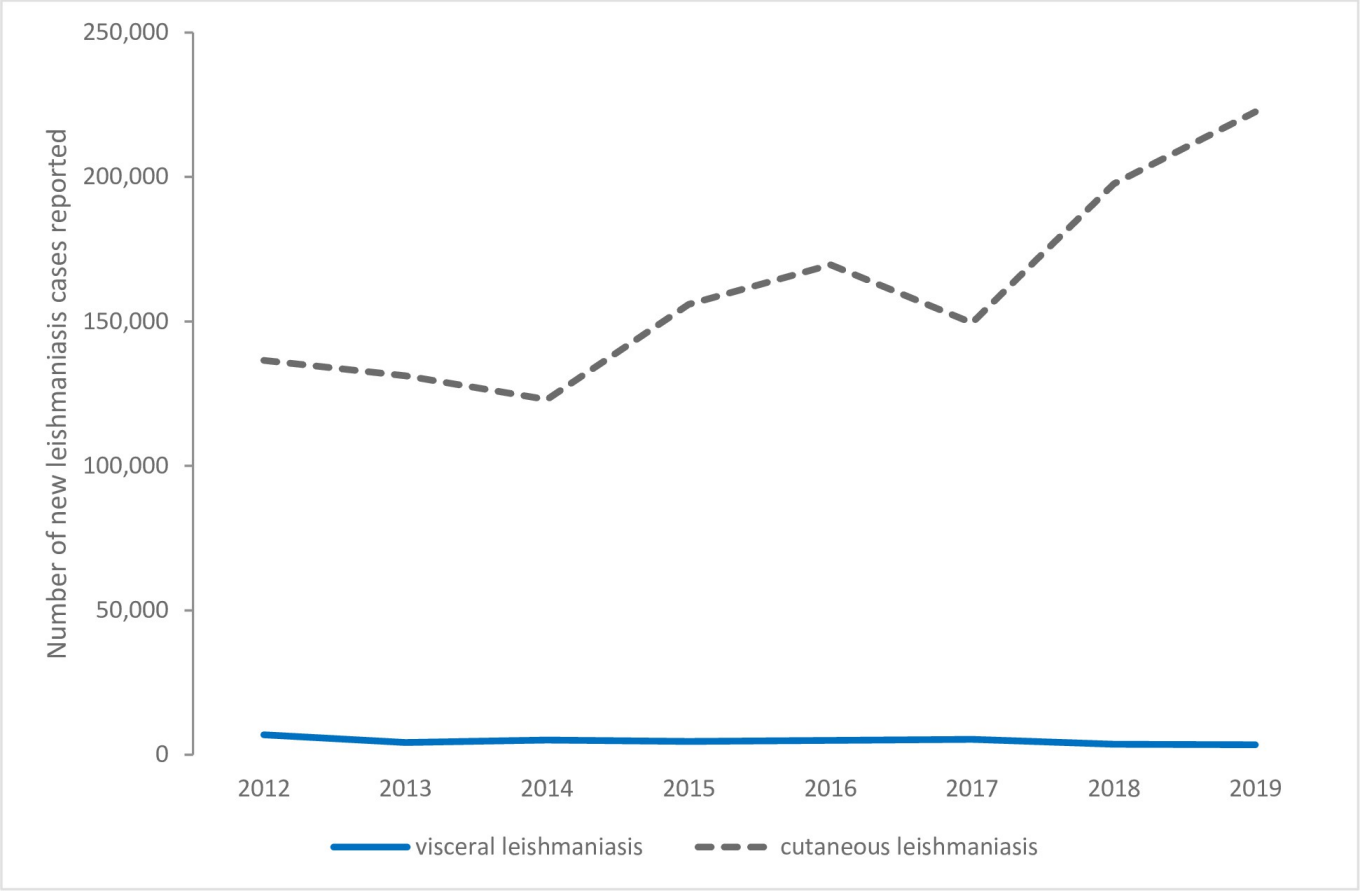
The number cutaneous and visceral leishmaniasis cases reported in the Eastern Mediterranean Region, 2012–2019, Global Health Observatory data repository (https://apps.who.int/gho/data/node.main.A1638?lang=en).

In 2019, 222,561 cutaneous leishmaniasis cases were reported in EMR. This represented an increase of 63% from the 136,470 reported in 2012. Most countries reported an increase, largest in Pakistan (from 6,598 in 2012 to 53,574 in 2019), Afghanistan (from 33,894 in 2012 to 55,225 in 2019), and Syria (from 55,894 in 2012 to 71,704 in 2019). There were some examples of declines (e.g., Iran from 20,947 to 8,161; S4 Table). Of the cases reported in 2019, 81% were reported in Syria (71,704; 32%), Afghanistan (55,225; 25%), and Pakistan (53,574; 24%). Of the remaining countries, 8 reported between 1,000 and 8,000 cases, 5 reported between 0 and 169 cases, and 3 did not report in 2019 (17 countries reporting; Fig 2).

Four countries reported 92% of the 3,450 visceral leishmaniasis cases in EMR in 2019: Sudan (2,563; 74%); Somalia (293; 8%); Iraq (170; 5%); and Yemen (132; 4%). Of the remaining 15 countries, 9 reported between 1 and 91 cases and 6 reported 0 cases in 2019. The number of cases in 2019 represented a 50% decline from 6,908 cases reported in 2012 (17 countries reporting; Fig 2). This decline was reflected in all countries except for small increases of less than 170 cases observed in Libya, Syria, and Yemen (S5 Table).

Rabies caused between 32 and 54 deaths each between 2012 and 2017, with a peak of 132 cases reported in 2013. The number of countries reporting deaths varied between 7 (2014) and 13 (2015 and 2017). Only 3 countries (Bahrain, Kuwait, and Qatar) consistently reported no deaths from rabies (S6 Table). Eight countries in EMR report mycetoma data with the largest number reported in Sudan in 2018 (633 cases) (S7 Table).

### Review of NTDs amenable to preventive chemotherapy

Lymphatic filariasis affected 3 countries in the EMR: Egypt, Sudan, and Yemen. Egypt and Yemen have eliminated lymphatic filariasis as a public health problem, in 2018 [9] and 2019, respectively [10]. Sudan implemented mass drug administration (MDA) in 2013, interrupted it and resumed in 2016, with an increase in coverage from 7% in 2016 to 41% in 2019 (Tables 4 and S8). Onchocerciasis remains endemic in Sudan and Yemen. Both countries increased MDA coverage, from 19% in 2018 to 87% in 2019 in Sudan and from 0% in 2017 to 93% in 2019 in Yemen (Table 4). Schistosomiasis affected 4 countries in EMR (Egypt, Somalia, Sudan, and Yemen). All countries achieved the target of reaching 75% of school-aged children (SAC), reaching 100% MDA coverage of SAC in Yemen (2013), 100% in Egypt (2014), 100% in Somalia (2018), and 95% in Sudan (2018) (S9 Table).

**Table 4:** National coverage of populations requiring prevention chemotherapy for lymphatic filariasis, onchocerciasis, schistosomiasis, STH**, and trachoma, PCT data portal, 2012–2019.

		Year of programme							
Disease	Country	2012	2013	2014	2015	2016	2017	2018	2019
**Lymphatic filariasis**									
	Sudan	0%	2%	0%	0%	7%	15%	17%	41%
**Onchocerciasis**									
	Sudan	0%	41%	0%%	40%	53%	92%	19%	87%
	Yemen	ND	ND	ND	ND	50%	0%	88%	93%
**Schistosomiasis**									
	Egypt	100%	100%	100%	95%	100%	96%	0%	100%
	Somalia	ND	ND	ND	ND	ND	36%	99%	0%
	Sudan	1%	25%	4%	38%	38%	32%	73%	38%
	Yemen	21%	78%	57%	89%	40%	100%	85%	79%
**STH**									
	Afghanistan	100%*	70%†	100%*	100%*	97%*/ 77%†	100%*/80%†	77%†	44%*/ 75%†
	Djibouti	ND	ND	100%*	100%*	ND	ND	ND	ND
	Iraq	ND	ND	ND	ND	100%†	ND	ND	ND
	Pakistan	ND	92%*	91%*	98%*	ND	95%*	ND	77%*/ 17%†
	Somalia	ND	ND	30%*	ND	ND	49%†	61%†	100%*
	Sudan	ND	1%†	3%†	25%†	11%†	ND	22%†	22%†
	Syria	ND	ND	ND	ND	ND	70%†	92%†	92%†
	Yemen	14%†	86%†	77%†	81%†	25%†	8%†	28%†	45%†
**Trachoma**									
	Afghanistan	ND	ND	ND	ND	ND	ND	ND	1%
	Egypt	ND	ND	ND	ND	ND	ND	ND	15%
	Pakistan	ND	ND	ND	ND	ND	46%	0%	0%
	Sudan	ND	8%	20%	31%	36%	60%	51%	52%
	Yemen	ND	ND	0%	0%	0%	0%	24%	0%

**Data reported only for first round in year.

*Preschool-aged children.

†School-aged children.

ND, no data.

Eight countries required PC for STH in EMR (Afghanistan, Djibouti, Iraq, Pakistan, Somalia, Sudan, Syria, and Yemen). Of the 8 countries, 3 required priority public health action as the last reported national coverage among SAC did not reach 75%: Somalia (61% SAC coverage, 2018), Sudan (22% SAC coverage, 2019), and Yemen (45% SAC coverage, 2019) (Table 4).

In 2019, 5 countries in EMR required ongoing interventions for elimination of trachoma as a public health problem (Afghanistan, Egypt, Pakistan, Sudan, and Yemen), 2 may require interventions with suspected trachoma-endemic areas (Libya and Somalia), and 3 countries need to be validated for having eliminated trachoma as a public health problem (Iraq, Saudi Arabia, and Tunisia). WHO validated Iran (2018), Morocco (2016), and Oman (2012) for having eliminated trachoma as a public health problem.

### Review provision of safe water, sanitation, and hygiene

In 2017, WHO/UNICEF joint monitoring programme reported the proportion of populations with access to safe water and appropriate sanitation. Among the 11 countries in EMR that reported on the population having access to safely managed drinking water, 4 countries (Iraq, Lebanon, Morocco, and Pakistan) had less than 60% of the population accessed it. Ten countries reported on access to handwashing facilities. Among them, 5 countries reported that 60% or less accessed it (Afghanistan, Pakistan, Somalia, Sudan, and Yemen). Among the 14 countries that reported on safely managed sanitation services, 5 countries (Djibouti, Iraq, Lebanon, Libya, and Morocco) had 60% or less people having access to it. Twenty-one countries reported on practicing open defecation, among them 7 countries reported more than 1% of the population practicing it (Afghanistan, Djibouti, Morocco, Pakistan, Somalia, Sudan, and Yemen; Table 5).

**Table 5:** Percentage populations with access to safe water and appropriate sanitation, Global Indicator Data Platform for Sustainable Development Goals, 2017.

	Water		Hygiene	Sanitation		
Country	Basic drinking water services	Safely managed drinking water services	Basic handwashing facilities on premises	Basic sanitation services	Safely managed sanitation services	practicing open defecation
Afghanistan	67	ND	38	43	ND	13
Bahrain	1	99	ND	4	96	0
Djibouti	75	ND	ND	27	36	17
Egypt	99	ND	90	33	61	0
Iran (Islamic Republic)	3	92	ND	88	ND	ND
Iraq	38	59	95	53	41	<1
Jordan	5	94	ND	17	81	<1
Kuwait	0	100	ND	0	100	0
Lebanon	45	48	ND	77	22	0
Libya	99	ND	ND	74	26	0
Morocco	17	70	ND	50	39	7
Oman	2	90	97	100	ND	0
Pakistan	56	35	60	60	ND	10
Palestine	97	ND	ND	36	61	<1
Qatar	3	96	ND	4	96	0
Saudi Arabia	100	ND	ND	22	78	0
Somalia	52	ND	10	38	ND	28
Sudan	60	ND	23	37	ND	24
Syrian Arab Republic	97	ND	71	91	ND	<1*
Tunisia	4	93	79	13	78	0
United Arab Emirates	98	ND	ND	2	96	<1
Yemen	63	ND	50	59	ND	20

*2016 data.

ND, no data.

## Discussion

Since the first roadmap for overcoming the impact of NTD [4], the EMR has made progress and achieved some successes. The region awaits certification of disease-free status for both dracunculiasis and the endemic treponematoses. Elimination of lymphatic filariasis as a public health problem has been validated in 2 countries (Egypt and Yemen), and elimination of trachoma as a public health problem has been validated in 3 (Iran, Morocco, and Oman). There has also been a 50% reduction in the annual number of visceral leishmaniasis cases reported.

Still substantial challenges remain in the prevention and control of NTDs. Among the NTDs prioritized for disease management, leishmaniasis remains a major public health problem in the EMR. It is endemic in 18 of the 22 countries in the region. Our rating identified a need for priority health action for cutaneous leishmaniasis in 11 countries, reflecting the increase in cases reported since 2012. Increases in cutaneous leishmaniasis have been attributed to ongoing conflicts [11], especially in Syria [12] and Afghanistan [13], population migration, lack of tools for vector and reservoir host control, lack of easy to administer safe and effective medicines, and inadequate funding support. Similar factors could also lead to an increase in visceral leishmaniasis and associated mortality. Although a framework for action on cutaneous leishmaniasis identified key objectives and targets to address the increase of cases in the region [14], this was not implemented with coordinated action. The Interregional meeting on leishmaniasis among neighbouring endemic countries in the Eastern Mediterranean, African, and European regions summarized major challenges including lack of access to treatment, absence of medicines donations, weak surveillance and underreporting, limited funding support, and inadequate infrastructure for vector control and surveillance. Recommendations also highlighted the need for mapping at subnational levels, especially in bordering areas, to inform both national and local coordination [15].

Despite the decline in the estimated population requiring interventions for NTDs, in 2018, there were still an estimated 78 million persons requiring interventions for NTDs in EMR [13]. In spite of successes in the delivery of PC programmes in many countries, others struggle with problems that result in suboptimal coverage. Nine countries in the region needed to continue MDA to control and prevent NTDs, of which 6 were rated as requiring priority action for 1 or more of the 5 NTDs prioritised for PC (i.e., Afghanistan, Egypt, Pakistan, Somalia, Sudan, and Yemen). Sudan is endemic for all the 5 diseases amenable to PC. A major challenge for MDA programmes is the management of the supply chain of medicines, which requires coordination between partners [16]. Successful programmes have adopted flexible approaches to work with new partners and empower affected communities [17].

For leprosy, the other NTD targeted for improved disease management, the prevalence has not declined in the 5 very affected countries. In Somalia, the national leprosy programme had been disrupted during the civil war and again reorganized in 2015. Resumption of active leprosy case-finding targeting most vulnerable population (e.g., internally displaced population living in concentrated camps) with high geographical coverage may explain the large increase of reported cases since then. These activities were supported by donor funds and complemented with training of health staff, prompt treatment with multidrug therapy, and mass awareness campaigns to address stigma [18].

Improving access to safe water, sanitation, and hygiene contributes to the prevention, control, and care of all NTDs to differing extents [19]. Safe water supply, hygiene measures, and improved sanitation can prevent diseases such as trachoma [20], STH [21], and schistosomiasis [22]. Environmental and waste management can control the breeding sites and habitats of vectors [23]. WASH interventions can also reduce the severity of disabilities associated with leprosy and lymphatic filariasis [24]. Additionally, WASH was a fundamental intervention under of each of the 4 components of under the BEST framework (behavior, environment, social inclusion, and treatment and care) used to outline all the interventions and programmatic aspects needed to tackle NTDs [25]. However, progress since 2012 has been limited, weakening the impact of interventions to prevent and control NTDs. Countries cannot separate problems they face in prevention and control of NTDs from socioeconomic contexts [26]. The socioeconomic status of countries is a key determinant of safe water and sanitation as well as universal health coverage. EMR progress stalled on the SDG to eradicate extreme poverty [27]. According to the World Bank, the number of people living under extreme poverty has increased in the Middle East and North Africa by 211% from 2012 to 2017 [28]. Yet, the failure to address NTDs cost developing communities the equivalent of billions of dollars each year, in direct health costs, loss of productivity, and reduced socioeconomic and educational attainment [29,30,31,32]. NTDs also place considerable financial strain on patients and their families [3]. Up to 75% of households affected by visceral leishmaniasis in Bangladesh, India, Nepal, and Sudan experience catastrophic costs while seeking diagnosis and treatment, even when tests and medicines are nominally free of charge [33,34,35,36]. Estimates suggest that achieving the WHO roadmap 2020 targets for leprosy and visceral leishmaniasis alone would increase productivity in EMR by nearly $5 billion dollars and avoid out-of-pocket expenses that push individuals further into poverty [37].

Robust healthcare systems and universal health coverage are needed to deliver both PC and intensified disease management for NTDs. Where health systems are weak, interventions such as MDA can become stand-alone programmes leading to inefficiencies [38]. These programmes need to be integrated into local health systems at the primary healthcare level, as these provide all necessary components and serve all communities, including those in remote rural areas. Integration of NTDs is cost-effective, can increase treatment coverage [39], and can lead to health systems strengthening [40]. As they target the most impoverished communities, NTD interventions need to be an integral part of achieving universal health coverage and will serve as a litmus test for its success [1,41]. Coordination with local government can deliver cross-sectoral action, such as improved sanitation [1].

Civil unrest and conflict worsen poverty, force displacement, and damage local structures, including healthcare systems. These consequences exacerbate NTDs [42]. In EMR, many countries have been defined as unstable [43]. EMR has been ranked as the least peaceful region [44]. Lack of political stability and continuous donor commitment contribute to lack of sustainable funding [45]. Nonetheless, countries have succeeded in delivering key achievements in the control of NTDs. The elimination of lymphatic filariasis as a public health problem in Yemen in 2019 during a period of military conflict and instability is a living example of this paradox [10]. The National Lymphatic Filariasis Elimination Programme was fully integrated with the National Leprosy Elimination Programme and made excellent progress due to strong collaboration with national and international partners, intensive training, mobilization of endemic communities, and close supervision [7].

This analysis is limited by the current absence of some proposed indicators to monitor progress towards key disease control targets (e.g., proportion of soil-transmitted helminth infections of moderate and heavy intensity) [3]. We presented data on 3 of the 5 pillars for the prevention and control of NTDs (disease management, PC, and access to safe water and sanitation). Data for PC rely on accurate population data to estimate the population at risk requiring treatment and correct tallying and aggregation of treatment records from community drug distributors to national levels. As such, these data are prone to errors especially where information systems are weak [46]. WHO develops standardized tools and methodologies to minimize common errors and supports countries in conducting data quality assessments such as coverage evaluation surveys, which can better determine true population coverage [47,48]. As such, the data presented in this review can be used for decision-making [49]. Limitations to population data still remain and can be obstacle when monitoring these interventions; hence, approaches are being explored such as geostatistical mapping [50] and the use independent sources of subnational data such as Demographic and Health Surveys [51]. Information on vector control and veterinary public health were not included due to lack of region-specific data; however, cross-cutting approaches such as One Health are important for NTDs as they recognize the dynamics between human, animal, and environmental health. WHO’s NTD Roadmap companion document on One Health emphasizes the need to involve multiple stakeholders, identify positive synergies, and support this paradigm shift to achieve the Roadmap targets. This is especially important for NTDs with significant zoonotic or environmental elements such as rabies, dengue, chikungunya, taeniasis, schistosomiasis, echinococcosis, food-borne trematodes, dracunculiasis, and Chagas [52]. Furthermore, data on the quantity of treatments delivered were unavailable at country level for some key diseases (e.g., leishmaniasis). We have employed routine surveillance data, which may underestimate the burden of disease [12].

Overall, EMR countries made progress in the prevention and control of NTDs, but challenges remain for specific countries and diseases. To achieve the 2030 targets set out in the new Roadmap [WHO 2020b], concerted action will be imperative over the next decade to capitalize over the demonstrated gains and overcome the persistent critical gaps [41,1,53]. The Regional Implementation Plan aims to coordinate, monitor, and accelerate these actions over the next 5 years through targeted milestones [54]. To sustain these efforts going forward, countries should evaluate their own situation, prioritize NTDs within national agendas, and explore more resilient and sustainable approaches to control, eliminate, or eradicate them.

### Key learning points

- Neglected tropical diseases (NTDs) are diseases of poverty that impose a devastating human, social, and economic burden on people living in predominantly tropical and subtropical areas.
- Increasing accountability for impact, intensifying cross-cutting approaches, and ownership of programmes by countries are key approaches to tackle NTDs.
- Countries with higher burden of NTDs are mostly with poor economic conditions; therefore, external donor support is critical to reach NTD targets.
- Routine conduct of neglected diseases’ programme reviews assists the countries to identify gaps and address them timely; during the programme reviews, special attention should be given to ensure data quality.
- Strong political commitment and partner support have proven that countries can achieve elimination of NTDs amidst complex emergency situations.

### Top five papers

1. Malecela M. Reflections on the decade of the neglected tropical diseases. International Health. 2019;11(5):338–340.
2. Engels D, Zhou X-N. Neglected tropical diseases: an effective global response to local poverty-related disease priorities. Infect Dis Poverty. 2020;9(1):10.
3. Al-Kubati AS, Al-Samie AR, Al-Kubati S, Ramzy RMR. The story of Lymphatic Filariasis elimination as a public health problem from Yemen. Acta Trop. 2020;212(105676):105676.
4. Muhjazi G, Gabrielli AF, Ruiz-Postigo JA, Atta H, Osman M, Bashour H, et al. Cutaneous leishmaniasis in Syria: A review of available data during the war years: 2011–2018. PLoS Negl Trop Dis. 2019;13(12):e0007827.
5. World Health Organization. Ending the neglect to attain the Sustainable Development Goals A road map for neglected tropical diseases 2021–2030. WHO; Geneva 2020.

## Supporting information

S1 Table WHO interventions for neglected tropical diseases endemic in the Eastern Mediterranean Region. (DOCX)

S2 Table The reported number of people requiring interventions against neglected tropical diseases in EMR by country, Global Indicator Data Platform for Sustainable Development Goals, 2012–2018. (DOCX)

S3 Table The number of new leprosy cases registered in EMR countries during 2012–2019, Global Health Observatory. (DOCX)

S4 Table The number of autochthonous cutaneous leishmaniasis cases reported globally and in EMR by country, 2012–2019, Global Health Observatory. (DOCX)

S5 Table The number of autochthonous visceral leishmaniasis cases reported globally and in EMR by country, 2012–2019, Global Health Observatory. (DOCX)

S6 Table The number of rabies deaths reported in EMR by country, 2012–2017, Global Health Observatory. (DOCX)

S7 Table The number of reported cases of mycetoma reported in EMR by country, 2016–2018, EMR Regional Health Observatory (2020). (DOCX)

S8 Table Geographical, program, and national coverage of drug treatment for lymphatic filariasis in the Eastern Mediterranean Region, 2016–2019, Preventive Chemotherapy Data Portal. (DOCX)

S9 Table Number school-aged children (SAC) requiring treatment for schistosomiasis and SAC national coverage achieved by treatment programs in four EMR countries (Egypt, Somalia, Sudan, and Yemen) 2012–2019, Preventive Chemotherapy Data Portal. (DOCX)
