# Supplementary material for: Review of the neglected tropical diseases programme implementation during 2012–2019 in the WHO-Eastern Mediterranean Region
Source: PLoS Negl Trop Dis. 2022 Sep 29;16(9):e0010665. doi: 10.1371/journal.pntd.0010665 (PMC9521802; doi:10.1371/journal.pntd.0010665)
Supplement: S1 Table — (DOCX) [file pntd.0010665.s001.docx]

# Supplementary information

**S1 Table:** WHO interventions for neglected tropical diseases endemic in the Eastern Mediterranean Region

| **Disease** | **Intervention** | **Target population** |
| --- | --- | --- |
| Leishmaniasis (cutaneous + visceral) | Individual case management; vector and animal reservoir control |  |
| Leprosy | Individual case management; contact-tracing, active case-finding, post-exposure prophylaxis |  |
| Rabies | Post-exposure prophylaxis, mass dog vaccination |  |
| Trachoma | SAFE (Surgery for trachomatous trichiasis, mass antibiotic treatment, facial cleanliness, environmental Improvement) |  |
| Helminthiases | Preventive chemotherapy | Preschool aged children, school aged children and at-risk adults |
| Mycetoma | Individual case management |  |
| Schistosomiasis | Preventive chemotherapy | School-aged children and at-risk adults in endemic areas |
| Onchocerciasis | Preventive chemotherapy and vector control | Entire at-risk population |
| Lymphatic filariasis | Preventive chemotherapy, morbidity management and disability prevention through essential package of care and vector control | Mass drug administration for entire at-risk population |
| Guinee Worm Disease | Individual case management |  |
