# Supplementary material for: Review of the neglected tropical diseases programme implementation during 2012–2019 in the WHO-Eastern Mediterranean Region
Source: PLoS Negl Trop Dis. 2022 Sep 29;16(9):e0010665. doi: 10.1371/journal.pntd.0010665 (PMC9521802; doi:10.1371/journal.pntd.0010665)
Supplement: S2 Table — (DOCX) [file pntd.0010665.s002.docx]

# Supplementary information

**S2 Table:** The reported number of people requiring interventions against neglected tropical diseases in EMR by country, Global Indicator Data Platform for Sustainable Development Goals, 2012-2018 [1]

|  | **Year of estimate** | | | | | | |  |
| --- | --- | --- | --- | --- | --- | --- | --- | --- |
| **Country** | **2012** | **2013** | **2014** | **2015** | **2016** | **2017** | **2018** | **2019** |
| Afghanistan | 14,045,789 | 13,291,651 | 13,384,908 | 13,765,392 | 13,275,429 | 14,786,722 | 14,834,701 | 16,222,053 |
| Bahrain | 0 | 0 | 0 | 6 | 3 | 2 | 1 | 5 |
| Djibouti | 131,879 | 105,768 | 107,378 | 108,810 | 110,561 | 110,561 | 110,561 | 110,561 |
| Egypt | 24,000,000 | 32,563,245 | 62,111 | 1,640,557 | 1,673,826 | 5,021,586 | 5,066,393 | 6,894,411 |
| Iran | 21,068 | 16,161 | 16,076 | 18,692 | 14,597 | 12,299 | 15,581 | 8,251 |
| Iraq | 3,574,492 | 2,003,182 | 2,040,539 | 2,107,072 | 2,170,486 | 2,170,486 | 2,170,486 | 2,170,486 |
| Jordan | 103 | 146 | 182 | 70 | 126 | 160 | 151 | 70 |
| Kuwait | 20 | 32 | 12 | 0 | 6 | 6 | 13 | 0 |
| Lebanon | 3 | 2 | 2 | 6 | 1 | 1 | 0 | 2 |
| Libya | 1,504 | 517 | 524 | 1,640 | 2,680 | 2,834 | 3,016 | 6,774 |
| Morocco | 3,034 | 2,741 | 2,665 | 2,917 | 5,014 | 6,936 | 40 | 5,576 |
| Oman | 10 | 16 | 18 | 5 | 0 | 1 | 1 | 80 |
| Pakistan | 31,511,018 | 30,292,389 | 30,418,380 | 31,056,287 | 31,683,212 | 31,683,212 | 31,683,212 | 25,234,450 |
| Qatar | 24 | 26 | 48 | 26 | 36 | 21 | 0 | 22 |
| Saudi Arabia | 1,476 | 2,001 | 2,207 | 1,496 | 1,354 | 1,021 | 940 | 1,113 |
| Somalia | 4,259,261 | 4,766,354 | 4,890,603 | 5,015,936 | 5,163,752 | 2,532,411 | 2,286,299 | 2,286,299 |
| Sudan | 30,750,805 | 28,227,078 | 28,461,970 | 26,533,962 | 25,572,281 | 11,031,353 | 11,930,091 | 12,015,065 |
| Syria | 55,916 | 72,033 | 53,912 | 50,998 | 47,406 | 1,925,000 | 1,767,108 | 2,440,286 |
| Tunisia | 277,152 | 200,856 | 3,415 | 6,648 | 6,087 | 4,800 | 6,650 | 7,085 |
| UAE | 0 | 31 | 0 | 0 | 40 | 0 | 0 | 55 |
| Yemen | 16,286,837 | 15,235,653 | 15,454,305 | 5,840,695 | 5,985,649 | 7,342,169 | 12,339,767 | 10,471,813 |
| **EMR Total** | **124,920,391** | **126,779,882** | **94,899,255** | **86,151,215** | **85,712,546** | **76,631,581** | **82,215,011** | **77,874,457** |

**References**

1. United Nations [Internet] SDG Indicators Database. Available from: <https://unstats.un.org/sdgs/dataportal/database>
