# Supplementary material for: Review of the neglected tropical diseases programme implementation during 2012–2019 in the WHO-Eastern Mediterranean Region
Source: PLoS Negl Trop Dis. 2022 Sep 29;16(9):e0010665. doi: 10.1371/journal.pntd.0010665 (PMC9521802; doi:10.1371/journal.pntd.0010665)
Supplement: S3 Table — (DOCX) [file pntd.0010665.s003.docx]

# Supplementary information

**S3 Table:** The number of new leprosy cases registered in EMR countries during 2012-2019, Global Health Observatory[1]

|  | **Year of Report** | | | | | | | |
| --- | --- | --- | --- | --- | --- | --- | --- | --- |
| **Country** | **2012** | **2013** | **2014** | **2015** | **2016** | **2017** | **2018** | **2019** |
| Afghanistan | 43 | 44 | 38 | 42 | 48 | 45 | 38 | 11 |
| Bahrain | ND | ND | ND | ND | 0 | 0 | 0 | ND |
| Djibouti | 14 | ND | ND | 0 | ND | ND | ND | ND |
| Egypt | 625 | ND | 538 | 598 | 721 | 735 | 407 | 540 |
| Iran (Islamic Republic) | 22 | 19 | 21 | 16 | 13 | 17 | 18 | 12 |
| Iraq | 1 | 3 | 3 | 0 | ND | ND | ND | ND |
| Jordan | 0 | 0 | 0 | 0 | 0 | 0 | 0 | 0 |
| Kuwait | 0 | 0 | 0 | ND | 6 | 6 | 0 | ND |
| Lebanon | 1 | ND | 3 | 4 | 5 | 5 | 1 | 1 |
| Libya | 4 | ND | 6 | 9 | 9 | 1 | ND | 2 |
| Morocco | 37 | 37 | 30 | 34 | 36 | 17 | ND | 23 |
| Oman | 3 | 5 | 6 | 2 | 0 | 1 | 1 | 0 |
| Palestine | 0 | 0 | ND | 0 | 0 | 0 | ND | ND |
| Pakistan | 764 | 657 | 586 | 532 | 514 | 502 | 437 | 438 |
| Qatar | 13 | 24 | 24 | 24 | 48 | ND | ND | 14 |
| Saudi Arabia | 2 | 4 | 2 | 3 | 7 | 9 | 22 | 25 |
| Somalia | 139 | ND | 14 | 107 | 674 | 1576 | 2610 | 2455 |
| Sudan | 846 | 1386 | 451 | 1117 | 630 | 1122 | 1109 | 1065 |
| Syrian Arab Republic | ND | ND | 34 | 0 | 0 | 5 | 2 | 0 |
| Tunisia | 0 | 0 | 0 | 2 | 1 | 2 | ND | 0 |
| United Arab Emirates | ND | 0 | ND | ND | 0 | 0 | 0 | ND |
| Yemen | 414 | 425 | 456 | 375 | 419 | 363 | 451 | 358 |
| **Total EMR** | **2928** | **2604** | **2212** | **2865** | **3131** | **4406** | **5096** | **4944** |

ND: no data

**References**

1. World Health Organization [Internet] Global Health Observatory - Neglected Tropical Diseases – Leprosy. Available from: <https://www.who.int/data/gho/data/themes/topics/leprosy-hansens-disease>
