# Supplementary material for: Review of the neglected tropical diseases programme implementation during 2012–2019 in the WHO-Eastern Mediterranean Region
Source: PLoS Negl Trop Dis. 2022 Sep 29;16(9):e0010665. doi: 10.1371/journal.pntd.0010665 (PMC9521802; doi:10.1371/journal.pntd.0010665)
Supplement: S4 Table — (DOCX) [file pntd.0010665.s004.docx]

# Supplementary information

**S4 Table:** The number of autochthonous cutaneous leishmaniasis cases reported globally and in EMR by country, 2012-2019, Global Health Observatory [1]

|  | **Year of Report** | | | | | | | |
| --- | --- | --- | --- | --- | --- | --- | --- | --- |
| **Country** | **2012** | **2013** | **2014** | **2015** | **2016** | **2017** | **2018** | **2019** |
| Afghanistan | 33,894 | 23,621 | 19,065 | 29,392 | 34,912 | 32,065 | 38,407 | 55,225 |
| Djibouti | ND | ND | ND | 0 | 0 | 0 | 0 | 0 |
| Egypt | 1,260 | 464 | 1,444 | 2,243 | 643 | 566 | 1,161 | 1,811 |
| Iran | 20,947 | 16,054 | 16,024 | 18,607 | 14,536 | 12,208 | 15,485 | 8,161 |
| Iraq | 2,486 | 1,648 | 2,691 | 17,525 | 17,566 | 18,854 | 11,426 | 7,056 |
| Jordan | 103 | 146 | 182 | 70 | 126 | 155 | 150 | 69 |
| Kuwait | 4 | 14 | 2 | 0 | 7 | 1 | 4 | ND |
| Lebanon | 2 | 0 | 2 | 3 | 0 | 0 | 0 | 2 |
| Libya | 1,500 | 505 | 516 | 1,632 | 2,662 | 2,815 | 2,977 | 6,744 |
| Morocco | 2,877 | 2,592 | 2,555 | 2,809 | 4,903 | 6,802 | 11,834 | 5,455 |
| Oman | 2 | ND | 0 | 0 | ND | ND | 1 | 0 |
| Pakistan | 6,598 | 3,717 | 14,634 | 16,647 | 27,151 | 8,024 | 19,361 | 53,574 |
| Palestine | 228 | 220 | 352 | 387 | 199 | 260 | 199 | 166 |
| Saudi Arabia | 1,464 | 1,988 | 2,190 | 1,490 | 1,337 | 1,007 | 921 | 1,096 |
| Somalia | ND | ND | ND | ND | ND | ND | ND | ND |
| Sudan | 206 | 336 | 1,053 | 3,503 | 3,011 | 4,107 | 3,299 | ND |
| Syrian Arab Republic | 55,894 | 71,996 | 53,876 | 50,972 | 47,377 | 53,232 | 80,215 | 71,704 |
| Tunisia | 5,376 | 4,113 | 3,368 | 6,611 | 6,065 | 4,902 | 7,467 | 7,058 |
| Yemen | 3,629 | 3,823 | 5,000 | 4,063 | 9,120 | 4,525 | 4,763 | 4,440 |
| **EMR Total** | **136,470** | **131,237** | **122,954** | **155,954** | **169,615** | **149,523** | **197,670** | **222,561** |
| **Global Total** | **203,026** | **192,597** | **185,454** | **215,542** | **235,399** | **217,773** | **261,708** | **277,224** |

ND: no data

**References**

1. World Health Organization [Internet] Global Health Observatory - Neglected Tropical Diseases – Leishmaniasis. Available from: <https://www.who.int/data/gho/data/themes/topics/gho-ntd-leishmaniasis>
