# Supplementary material for: Review of the neglected tropical diseases programme implementation during 2012–2019 in the WHO-Eastern Mediterranean Region
Source: PLoS Negl Trop Dis. 2022 Sep 29;16(9):e0010665. doi: 10.1371/journal.pntd.0010665 (PMC9521802; doi:10.1371/journal.pntd.0010665)
Supplement: S5 Table — (DOCX) [file pntd.0010665.s005.docx]

# Supplementary information

**S5 Table:** The number of autochthonous visceral leishmaniasis cases reported globally and in EMR by country, 2012-2019, Global Health Observatory [1]

|  | **Year of Report** | | | | | | | |
| --- | --- | --- | --- | --- | --- | --- | --- | --- |
| **Country** | **2012** | **2013** | **2014** | **2015** | **2016** | **2017** | **2018** | **2019** |
| Afghanistan | 24 | 16 | 12 | 8 | 9 | 14 | 23 | 20 |
| Djibouti | ND | ND | ND | 10 | 9 | 34 | 12 | 1 |
| Egypt | 0 | 0 | 0 | 0 | 0 | 0 | 0 | 0 |
| Iran (Islamic Republic) | 94 | 81 | 26 | 59 | 61 | 60 | 67 | 72 |
| Iraq | 1,045 | 575 | 362 | 427 | 183 | 172 | 259 | 170 |
| Jordan | 0 | 0 | 0 | 0 | 0 | 0 | 1 | 1 |
| Lebanon | 0 | 2 | 0 | 0 | 0 | ND | 0 | 0 |
| Libya | ND | 12 | 1 | 0 | 10 | 18 | 34 | 28 |
| Morocco | 113 | 111 | 85 | 81 | 92 | 106 | 106 | 91 |
| Oman | 1 | ND | 0 | 1 | ND | ND | 0 | 0 |
| Pakistan | 14 | 7 | 7 | ND | ND | ND | ND | 0 |
| Palestine | 8 | 3 | 1 | 4 | 3 | 3 | 7 | 5 |
| Saudi Arabia | 8 | 5 | 10 | 3 | 4 | 4 | 1 | 0 |
| Somalia | 394 | 936 | 1045 | 1,165 | 734 | 857 | 413 | 293 |
| Sudan | 5,153 | 2,389 | 3,415 | 2,829 | 3,810 | 3,894 | 2,584 | 2,563 |
| Syrian Arab Republic | 17 | 30 | 36 | 20 | 25 | 55 | 38 | 47 |
| Tunisia | 37 | 38 | 44 | 30 | 17 | 23 | 19 | 27 |
| Yemen | ND | ND | 15 | 5 | ND | 44 | 56 | 132 |
| **EMR Total** | **6,908** | **4,205** | **5,059** | **4,642** | **4,957** | **5,284** | **3,620** | **3,450** |
| **Global Total** | **41,125** | **28,278** | **30,801** | **23,958** | **22,503** | **22,500** | **17,099** | **13,814** |

ND: no data

**References**

1. World Health Organization [Internet] Global Health Observatory - Neglected Tropical Diseases –Leishmaniasis. Available from: <https://www.who.int/data/gho/data/themes/topics/gho-ntd-leishmaniasis>
