# Supplementary material for: Review of the neglected tropical diseases programme implementation during 2012–2019 in the WHO-Eastern Mediterranean Region
Source: PLoS Negl Trop Dis. 2022 Sep 29;16(9):e0010665. doi: 10.1371/journal.pntd.0010665 (PMC9521802; doi:10.1371/journal.pntd.0010665)
Supplement: S6 Table — (DOCX) [file pntd.0010665.s006.docx]

# Supplementary information

**S6 Table:** The number of rabies deaths reported in EMR by country, 2012-2017, Global Health Observatory[1]

|  | **Year of report** | | | | | |
| --- | --- | --- | --- | --- | --- | --- |
| **Country** | **2012** | **2013** | **2014** | **2015** | **2016** | **2017** |
| Afghanistan | ND | ND | ND | ND | ND | ND |
| Bahrain | 0 | 0 | 0 | 0 | 0 | 0 |
| Djibouti | ND | ND | ND | ND | ND | ND |
| Egypt | 21 | 32 | ND | ND | ND | ND |
| Iran (Islamic Republic) | 6 | 5 | 4 | 7 | 1 | 12 |
| Iraq | 11 | 8 | 12 | 6 | ND | 9 |
| Jordan | ND | ND | ND | 0 | 1 | 5 |
| Kuwait | 0 | 0 | ND | 0 | 0 | 0 |
| Lebanon | ND | ND | ND | 2 | 0 | 1 |
| Libya | ND | ND | ND | ND | ND | ND |
| Morocco | 6 | 24 | 20 | 19 | 17 | 15 |
| Oman | ND | ND | ND | ND | ND | ND |
| Pakistan | ND | ND | ND | ND | ND | ND |
| Qatar | 0 | ND | ND | 0 | 0 | 0 |
| Saudi Arabia | 0 | ND | ND | 3 | 2 | 1 |
| Somalia | ND | ND | ND | 3 | 2 | 1 |
| Sudan | 2 | 2 | 1 | 0 | 1 | 1 |
| Syrian Arab Republic | 5 | 7 | 0 | 3 | 4 | 7 |
| Tunisia | 3 | 6 | 3 | 6 | 4 | 1 |
| United Arab Emirates | ND | ND | ND | ND | ND | ND |
| Yemen | ND | 48 | ND | ND | ND | ND |
| **EMR** | **54** | **132** | **40** | **49** | **32** | **53** |

ND: No data

**References**

1. World Health Organization [Internet] Global Health Observatory - Neglected Tropical Diseases – Rabies. Available from: <https://www.who.int/data/gho/data/themes/topics/rabies>
