# Supplementary material for: Review of the neglected tropical diseases programme implementation during 2012–2019 in the WHO-Eastern Mediterranean Region
Source: PLoS Negl Trop Dis. 2022 Sep 29;16(9):e0010665. doi: 10.1371/journal.pntd.0010665 (PMC9521802; doi:10.1371/journal.pntd.0010665)
Supplement: S7 Table — (DOCX) [file pntd.0010665.s007.docx]

# Supplementary information

**S7 Table:** The number of reported cases of mycetoma reported in EMR by country, 2016-2018, EMR Regional Health Observatory [1]

|  | **Year Report** | | |
| --- | --- | --- | --- |
| **Country** | **2016** | **2017** | **2018** |
| Lebanon | 0 | ND | ND |
| Libya | 5 | ND | ND |
| Oman | 0 | ND | ND |
| Palestine | 0 | ND | ND |
| Qatar | 0 | 0 | 0 |
| Saudi Arabia | 52 | ND | ND |
| Sudan | ND | ND | 633 |
| United Arab Emirates | ND | ND | 0 |

ND: No data

**References**

1. World Health Organization [Internet] Eastern Mediterranean Regional Office – Regional Health Observatory. Available from: <https://rho.emro.who.int/index.php/Indicator/TermID/64>
