# Supplementary material for: Review of the neglected tropical diseases programme implementation during 2012–2019 in the WHO-Eastern Mediterranean Region
Source: PLoS Negl Trop Dis. 2022 Sep 29;16(9):e0010665. doi: 10.1371/journal.pntd.0010665 (PMC9521802; doi:10.1371/journal.pntd.0010665)
Supplement: S8 Table — (DOCX) [file pntd.0010665.s008.docx]

# Supplementary information

**S8 Table:** Geographical, program and national coverage of drug treatment for lymphatic filariasis in the Eastern Mediterranean Region, 2016-2019, Preventive Chemotherapy Data Portal[1]

|  | **Year Program** | | |  |
| --- | --- | --- | --- | --- |
| **Measure** | **2016** | **2017** | **2018** | **2019** |
| Number of Intervention Units (IUs) requiring PC | 96 | 60 | 61 | 61 |
| Number of IUs covered | 12 | 11 | 16 | 43 |
| Geographical coverage | 13% | 18% | 26% | 71% |
| Total population of IUs | 2,982,383 | 1,869,017 | 2,185,864 | 6,252,911 |
| Reported number of people treated | 921,947 | 1,455,505 | 1,713,149 | 4,408,107 |
| Program (drug) coverage | 31% | 78% | 78% | 71% |
| National coverage | 7% | 15% | 17% | 41% |
| Number of lymphedema patients reported* | 1306 | | | |
| Number of hydrocele patients reported* | 18 | | | |

* Represents the cumulative number reported since 2007

**References**

1. World Health Organization [Internet] Neglected Tropical Diseases - Preventive Chemotherapy Data Portal. Available from: <https://www.who.int/data/preventive-chemotherapy>
