# Supplementary material for: Review of the neglected tropical diseases programme implementation during 2012–2019 in the WHO-Eastern Mediterranean Region
Source: PLoS Negl Trop Dis. 2022 Sep 29;16(9):e0010665. doi: 10.1371/journal.pntd.0010665 (PMC9521802; doi:10.1371/journal.pntd.0010665)
Supplement: S9 Table — (DOCX) [file pntd.0010665.s009.docx]

# Supplementary information

**S9 Table:** Number school-aged children (SAC) requiring treatment for schistosomiasis and SAC national coverage achieved by treatment programs in four EMR countries (Egypt, Somalia, Sudan and Yemen) 2012-2019, Preventive Chemotherapy Data Portal [1]

|  | **Year of Program** | | | | | | | | |  |
| --- | --- | --- | --- | --- | --- | --- | --- | --- | --- | --- |
|  | **2012** | | | **2013** | **2014** | **2015** | **2016** | **2017** | **2018** | **2019** |
| **Number to be treated** | |  | |  |  |  |  |  |  |  |
| Egypt | 60,126 | | | 61,120 | 62,111 | 70,000 | 71,426 | 2,927,480 | 2,953,601 | 3,722,983 |
| Somalia | 284,813 | | | 293,206 | 301,868 | 309,604 | 318,728 | 1,381,882 | 1,406,787 | 1,434,961 |
| Sudan | 2,238,014 | | | 4,665,241 | 4,763,522 | 4,870,608 | 4,987,941 | 3,984,541 | 4,953,929 | 4,516,705 |
| Yemen | 2,880,848 | | | 2,947,877 | 3,015,648 | 2,734,728 | 2,802,598 | 1,530,655 | 2,571,304 | 3,305,807 |
| **SAC National coverage** | | |  |  |  |  |  |  |  |  |
| Egypt | ND | | | 34% | 100% | 98% | 100% | 95% | 0% | 100% |
| Somalia | ND | | | ND | ND | ND | ND | 51% | 100% | 0% |
| Sudan | 3% | | | 17% | 3% | 58% | 24% | 34% | 95% | 55% |
| Yemen | 35% | | | 100% | 100% | 100% | 33% | 100% | 72% | 94% |

ND: No data

**References**

1. World Health Organization [Internet] Neglected Tropical Diseases - Preventive Chemotherapy Data Portal. Available from: <https://www.who.int/data/preventive-chemotherapy>
